# Wikidata as a semantic framework for the Gene Wiki initiative

**DOI:** 10.1093/database/baw015

**Published:** 2016-03-17

**Authors:** Sebastian Burgstaller-Muehlbacher, Andra Waagmeester, Elvira Mitraka, Julia Turner, Tim Putman, Justin Leong, Chinmay Naik, Paul Pavlidis, Lynn Schriml, Benjamin M Good, Andrew I Su

**Affiliations:** 1 The Scripps Research Institute, La Jolla, CA, USA; 2 micelio.be, Antwerp, Belgium; 3 University of Maryland Baltimore, Baltimore, MD, USA; 4 The University of British Columbia, Vancouver, British Columbia, Canada and; 5 Bangalore Inst. Of Technology, Visvesvaraya Technological University, Bangalore, Karnataka

## Abstract

Open biological data are distributed over many resources making them challenging to integrate, to update and to disseminate quickly. Wikidata is a growing, open community database which can serve this purpose and also provides tight integration with Wikipedia. In order to improve the state of biological data, facilitate data management and dissemination, we imported all human and mouse genes, and all human and mouse proteins into Wikidata. In total, 59 721 human genes and 73 355 mouse genes have been imported from NCBI and 27 306 human proteins and 16 728 mouse proteins have been imported from the Swissprot subset of UniProt. As Wikidata is open and can be edited by anybody, our corpus of imported data serves as the starting point for integration of further data by scientists, the Wikidata community and citizen scientists alike. The first use case for these data is to populate Wikipedia Gene Wiki infoboxes directly from Wikidata with the data integrated above. This enables immediate updates of the Gene Wiki infoboxes as soon as the data in Wikidata are modified. Although Gene Wiki pages are currently only on the English language version of Wikipedia, the multilingual nature of Wikidata allows for usage of the data we imported in all 280 different language Wikipedias. Apart from the Gene Wiki infobox use case, a SPARQL endpoint and exporting functionality to several standard formats (e.g. JSON, XML) enable use of the data by scientists.

In summary, we created a fully open and extensible data resource for human and mouse molecular biology and biochemistry data. This resource enriches all the Wikipedias with structured information and serves as a new linking hub for the biological semantic web.

**Database URL:** https://www.wikidata.org/

## Introduction

Wikipedia (www.wikipedia.org) is a well-established encyclopedia and collection of free form text, operated by the Wikimedia Foundation and edited by thousands of volunteer editors. As the seventh most-visited site on the Internet (http://www.alexa.com/topsites), Wikipedia has articles on a broad range of topics. With respect to molecular biology articles, at least two systematic efforts have been described, both initiated in 2007. The RNA Wikiproject created ∼600 new Wikipedia articles on non-coding RNA families (1). In parallel, our Gene Wiki team created a collection of ∼8000 Wikipedia articles on human genes (2). Since its inception, the Gene Wiki has grown into an integral and strongly interlinked part of the English Wikipedia, now counting >11 000 articles (3, 4). The Gene Wiki articles have been expanded by the Wikipedia community and are highly accessed by users of Wikipedia, collectively viewed >4 million times per month (according to http://stats.grok.se/en/).

Gene Wiki articles consist of two central parts, the free text and the Gene Wiki infobox. The free text represents a review of a gene's function, biological role and impact on human health and disease. The Gene Wiki infobox provides structured data on the human gene and protein, and the orthologous mouse gene and protein. The data in the infobox comprises standardized identifiers and chromosome coordinates as well as functional annotation with Gene Ontology terms (5), structural information from the Protein Data Bank (PDB) (6) and tissue-specific gene expression (7) (e.g. https://en.wikipedia.org/wiki/Reelin).

Wikipedia has proven a highly effective medium for collaboratively capturing unstructured text, but is technically lacking facilities for authoring structured data. Several attempts have been made to better represent structured data within Wikipedia (8, 9). In late 2012, the Wikidata project (www.wikidata.org) was launched with the goal of creating an open, structured knowledge repository to complement and facilitate the unstructured content in Wikipedia (10). Like all other Wikimedia projects, Wikidata can be edited by anyone, and maintains an extensive version history for every item to allow for easy comparisons or reversions to past states. All content in Wikidata is licensed under CC0 (https://creativecommons.org/about/cc0) and therefore can be used by anyone without restrictions.

**Figure 1:**
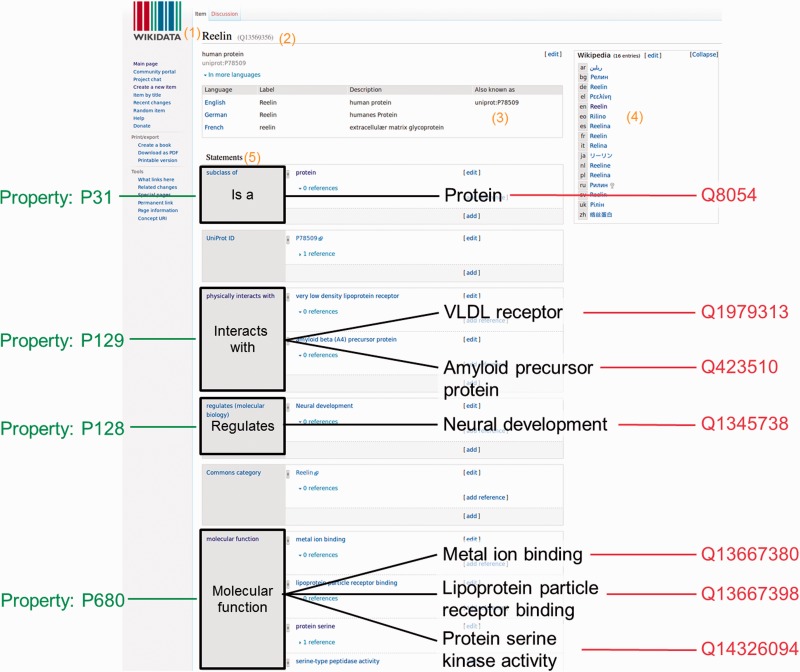
Wikidata item and data organization. Wikidata items can be added or edited by anyone manually. A Wikidata item consists of: (1) a language-specific label, (2) its unique identifier, (3) language specific aliases, (4) interwiki links to the different language Wikipedia articles or other Wikimedia projects and (5) a list of statements. For this specific example, the human protein Reelin was used (https://www.wikidata.org/wiki/Q13569356)

Wikidata consists of two entity types – items (e.g. https://www.wikidata.org/wiki/Q7474 for Rosalind Franklin) and properties (e.g. https://www.wikidata.org/wiki/Property:P351 for NCBI Entrez gene ID)—and every entity is assigned a unique identifier. Wikidata items and properties all have a label, a description and aliases (Figure 1). Every item record contains a list of claims in the form of triples. The subject of the triple is the Wikidata item on which the claim appears, the predicate is a Wikidata property, and the object is a date, a string, a quantity, a URL or another Wikidata item. For example, the claim that Rosalind Franklin received her Ph.D. at the University of Cambridge is represented as Q7474 (Rosalind Franklin) - P69 (educated at) - Q35794 (University of Cambridge). Claims can be further amended with qualifiers (to indicate the context in which the triple is valid), and references can be added to indicate the provenance of the claim. The overall combination of a claim and references is referred to as a ‘statement’. A full description of the Wikidata data model can be found at https://www.mediawiki.org/wiki/Wikibase/DataModel/Primer.

A primary motivation for creating Wikidata was enabling easy accessibility by all language-specific Wikipedias. Now, statements about any Wikidata item can be displayed in the context of any Wikipedia item. This process is facilitated by ‘interwiki links’ that establish connections between structured Wikidata items and the Wikipedia articles they are most closely related to. All major projects from the Wikimedia Foundation, including the language-specific Wikipedias, are linked to Wikidata using interwiki links. These links can be established between any existing Wikidata item and other MediaWiki content pages, e.g. Wikipedia articles. Currently, a single Wikidata item can have an interwiki link to many different language-specific Wikipedia articles on the same topic. In return, this setup allows for all linked language-specific Wikipedia articles to access data from the central Wikidata item. Importantly, a Wikipedia article in a certain language can only have one interwiki link to a Wikidata item and a Wikidata item can only be linked to one single Wikipedia article in a certain language. Of note, the interwiki links are also used to organize the links to other language versions of a Wikipedia article, visible in the sidebar of many Wikipedia articles. This system has the advantage that data are stored once in Wikidata, and then made available for reuse across the entire Wikimedia universe.

Wikidata enables programmatic querying and access outside of the Wikipedia context. Specifically, Wikidata offers a Representational State Transfer (REST) API to easily perform structured data queries and retrieve Wikidata statements in structured formats. In addition, more complex queries are possible via a SPARQL Protocol and RDF Query Language (SPARQL) endpoint (https://query.wikidata.org) and a custom-built WikiData Query (WDQ) tool (http://wdq.wmflabs.org/wdq/).

In this work, we describe our efforts to migrate our Gene Wiki bot from English Wikipedia to Wikidata. This system offers significant advantages with respect to maintainability of the data, accessibility within the Wikipedia ecosystem, and programmatic integration with other resources.

## Database construction and usage

**Figure 2:**
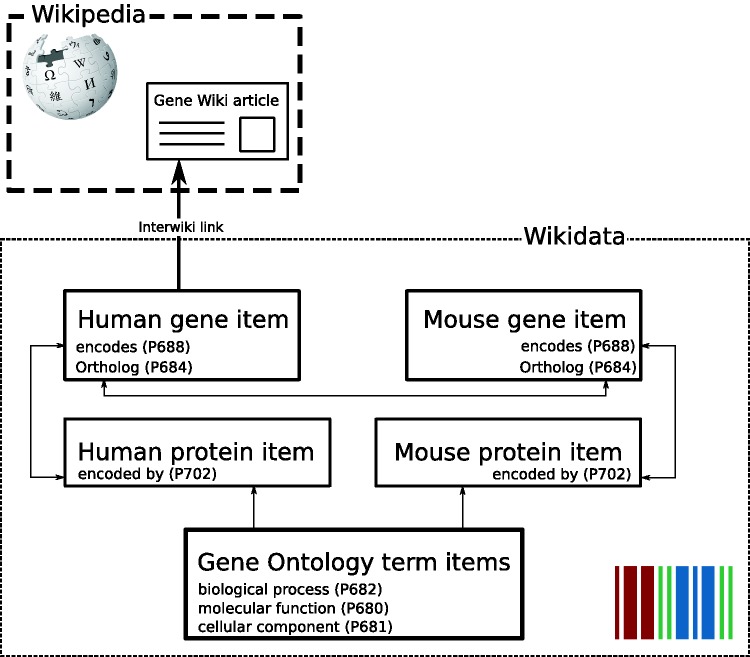
Gene Wiki data model in Wikidata. Each entity (human gene, human protein, mouse gene, mouse protein) is represented as a separate Wikidata item. Arrows represent direct links between Wikidata statements. The English language interwiki link on the human gene item points to the corresponding Gene Wiki article on the English Wikipedia.

In collaboration with the Wikidata community, we decided to implement the representation of genes and proteins in Wikidata as separate Wikidata items. These gene and protein items are linked by the reciprocal properties ‘encodes’ (P688) and ‘encoded by’ (P702) carried by genes and proteins, respectively (Figure 2). Furthermore, orthologous genes between species are reciprocally linked by the property ortholog (P684) and also link out to NCBI HomoloGene (11) with the HomoloGene ID (P593). Homologous genes in the HomoloGene database and therefore also on Wikidata gene items share the same ID and can also be associated this way. The community discussion and decision process to establish this model, a very crucial mechanism in Wikidata, as well as Wikipedia, can be viewed here: https://www.wikidata.org/wiki/Wikidata_talk:WikiProject_Molecular_biology.

**Figure 3:**
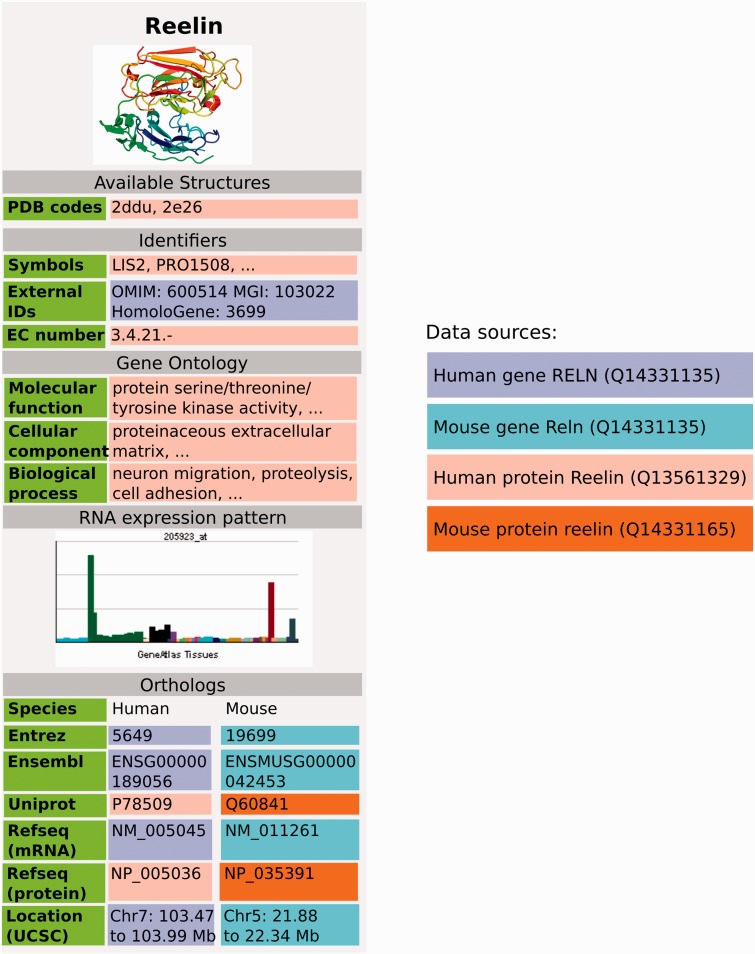
GeneWiki infobox populated with data from Wikidata, using data from Wikidata items Q414043 for the human gene, Q13561329 for human protein, Q14331135 for the mouse gene and Q14331165 for the mouse protein. Three dots indicate that there is more information in the real Gene Wiki infobox for Reelin (https://en.wikipedia.org/wiki/Reelin).

### Data model and data sources for Wikidata

We populated Wikidata with items for all *Homo sapiens* (human) genes, *Homo sapiens* proteins, *Mus musculus* (mouse) genes and *Mus musculus* proteins (Table 1). As described above, each gene and each protein was represented as a single Wikidata item. A full list of Wikidata properties used on gene and protein items is provided in Table 2.

**Table 1:** Overview on *Homo sapiens* and *Mus musculus* data imported to Wikidata

Data source	Item count
*Homo sapiens* genes (NCBI release 107)	59 721
*Homo sapiens* proteins (Uniprot)	27 306
*Mus musculus* genes (NCBI release 105)	73 355
*Mus musculus* proteins (Uniprot)	16 728
Gene Ontology terms	17 098

**Table 2:** Wikidata properties used in this study

Label	Target data type	Property ID	Description
Wikidata gene items			
Subclass of	Wikidata item	P279	Defines to what category this item belongs to. Every gene item carries the value ‘gene’ (Q7187). Further subcategories are protein coding gene (Q20747295), ncRNA gene (Q27087), snRNA gene (Q284578), snoRNA gene (Q284416), rRNA gene (Q215980), tRNA gene (Q201448) and pseudogene (Q277338).
Entrez Gene ID	String	P351	The NCBI gene ID as in annotation release 107
Found in taxon	Wikidata item	P703	The taxon, either *Homo sapiens* (Q5) or *Mus musculus* (Q83310)
Ensembl Gene ID	String	P594	Gene ID from the Ensembl database
Ensembl Transcript ID	String	P704	Transcript IDs from the Ensembl database
Gene symbol	String	P353	Human gene symbol according to HUGO Gene Nomenclature Committee
HGNC ID	String	P354	HUGO Gene Nomenclature Committee ID
HomoloGene ID	String	P593	Identifier for the HomoloGene database
NCBI RefSeq RNA ID	String	P639	
Chromosome	Wikidata item	P1057	Chromosome a gene is residing on
Ortholog	Wikidata item	P684	Ortholog based on the Homologene database
Genomic start	String	P644	Genomic start according to GRCh37 and GRCh38, sourced from NCBI
Genomic stop	String	P645	Genomic stop according to GRCh37 and GRCh38, sourced from NCBI
Mouse Genome Informatics ID	String	P671	Jackson lab mouse genome informatics database
encodes	Wikidata item	P688	Protein item a gene encodes
*Wikidata protein items*			
Subclass of	Wikidata item	P279	protein (Q8054)
UniProt ID	String	P352	
PDB ID	String	P638	Protein structure IDs from PDB.org
RefSeq protein ID	String	P637	NCBI RefSeq Protein ID
encoded by	Wikidata item	P702	Gene item a protein is encoded by
Ensembl protein ID	String	P705	
EC number	String	P591	Enzyme Category number
Protein structure image	Wiki Commons Media File	P18	Prefered protein structure image retrieved from PDB.org
Cell component	Wikidata item	P681	Gene ontology term items for cell components
Biological process	Wikidata item	P682	Gene ontology term items for biological processes
Molecular function	Wikidata item	P680	Gene ontology term items for molecular function

Column one contains the description as in Wikidata, column two the data type, column three the property number and column four a short description of the nature of the content.

Briefly summarized, for gene items, we imported data from the latest annotation releases from NCBI (*Homo sapiens* release 107, *Mus musculus* release 105) and created statements using many properties, including Entrez Gene IDs, RefSeq RNA IDs and chromosomal positions (11). Ensembl Gene IDs and Ensembl Transcript IDs were also imported and added to each gene item in Wikidata (12). Genes were categorized according to eight subclasses. A generic subclass was used to identify a Wikidata item as a gene (Q7187), for increased granularity, the subclasses protein coding gene (Q20747295), ncRNA gene (Q27087), snRNA gene (Q284578), snoRNA gene (Q284416), rRNA gene (Q215980), tRNA gene (Q201448) and pseudo gene (Q277338) were added (Table 2). Genomic coordinates were encoded using the properties chromosome (P1057), genomic start (P644), and genomic end (P645), and the qualifier property ‘GenLoc assembly’ (P659) was used to indicate the corresponding assembly version—GRCh37 (Q21067546) or GRCh38 (Q20966585). Gene symbols were added based on the HUGO Gene Nomenclature Committee (HGNC) and HGNC IDs were also added to each gene item (24). For mouse gene nomenclature, the Jackson Laboratory Mouse Genome Informatics (MGI) data were used (13).

For protein items, we used UniProt as the primary data source. All protein items received the ‘subclass of’ (P279) property value ‘protein’ (Q8054). A range of protein annotations were also added, including NCBI RefSeq Protein IDs (P637), Ensembl Protein IDs (P705), and PDB IDs (P638). Gene Ontology terms were introduced as separate Wikidata items, and annotations were added to protein items using three separate properties for Molecular Function (P680), Cell Component (P681) and Biological Process (P682).

### Data import and data maintenance

In order to implement a data model in Wikidata and to populate this data model with data, two types of community processes need to be passed. The properties required for the data model need to be proposed to and discussed by the community and if consensus can be reached, the property will be created in Wikidata. Furthermore, large scale data import to Wikidata can only be done with a bot user account, which has enhanced write permissions compared to a standard user account. Bot user accounts also go through a community approval process, in order to determine if they are performing beneficial tasks and if they are operating reliably. For the present project, we successfully proposed a series of properties required for our data model and also reached community consensus on the usefulness of our bots task.

We implemented the data importing process using Python (www.python.org) scripts, colloquially termed as bots by the Wikidata community. We run these bots with the Wikidata user account ProteinBoxBot (https://www.wikidata.org/wiki/User:ProteinBoxBot). The source code for the bots is available under GNU AGPLv3 (http://www.gnu.org/licenses/agpl.html) on our Bitbucket repository (https://bitbucket.org/sulab/wikidatabots/). As our aim was to establish and also to maintain a high quality data source, the bots are designed to update the data from their original sources on a regular basis. Bots are run every few days to weeks, depending on the data source, to ensure data being up-to-date. Before our bots write data to a Wikidata item or create a new item, thorough checks are being performed to ensure that the correct item has been selected for writing or an item does not yet exist and so a new one should be created.

Data on Wikidata can be edited by anybody. In order to keep our data model consistent and to detect accidental erroneous modification or potential vandalism of data, we implemented a set of consistency tests based on SPARQL queries (https://bitbucket.org/sulab/wikidatasparqlexamples/src). These queries detect inconsistencies in our data model and, as an ongoing effort, we implemented bots which execute these SPARQL queries and report and resolve the inconsistencies detected. In view of Wikidata being completely open, the number of inconsistencies we experienced so far is low.

As mentioned in the introduction, interwiki links are organized in Wikidata. Thus, users from the different language Wikipedias aim to link their articles to the same Wikidata item. The data model in Wikidata therefore needs to be designed in a way that all interwiki links unambiguously are present on the correct Wikidata item. Otherwise, Wikipedia users might be encouraged to merge items which are separate things in one's data model. The several dozens of merges we experienced where the biggest challenge to our data model so far. To prevent and resolve it, we implemented a bot which can detect and undo these merges. Since we also unified the interwiki links on the human genes, these merges have now decreased to zero, showing the importance of consistent interwiki links.

### Populating gene wiki infoboxes with data from wikidata

As a first use case of the data, we focused on using the gene and protein data imported into Wikidata to populate Wikipedia Gene Wiki infoboxes. In our data model, we connected Wikipedia Gene Wiki pages to Wikidata human gene items with interwiki links (Figure 2). Four Wikidata items are required to fully represent one Gene Wiki infobox on a Wikipedia article. For this paper, we chose the gene *RELN* (protein Reelin) as an example. Specifically, the English language Wikipedia page (https://en.wikipedia.org/wiki/Reelin) of Reelin is directly linked to the human gene Wikidata item (Q414043) with an interwiki link. The Wikidata human gene item in turn links to the human protein (Q13561329), mouse gene (Q14331135), and mouse protein (Q14331165) using the data model described in Figure 2.

In order for Wikipedia pages to retrieve data from Wikidata, we used the MediaWiki extension module Scribunto (https://www.mediawiki.org/wiki/Extension:Scribunto), which integrates scripting capabilities based on the programming language Lua (http://www.lua.org). We created a new module in Lua code which generates the entire Wikipedia Gene Wiki infobox based on Wikidata data (https://en.wikipedia.org/wiki/Module:Infobox_gene). Using this new Wikidata-based infrastructure, a Gene Wiki infobox can be added to any Wikipedia page for a human gene by simply adding the markup code ‘{{infobox gene}}’, provided the Wikipedia page has an interwiki link to a Wikidata human gene item. Not all data present on the Wikidata gene and protein items are currently also represented in the Gene Wiki info boxes, at this stage, we aimed at largely replicating the content of the previous, template based infoboxes. By clicking the Wikidata link in the sidebar or the infobox of a Gene Wiki page, a user can directly access the Wikidata item and view or modify the entire data available on the item. In order to display additional data in the infoboxes, slight adaptations of the Lua code are required. Of note, the Lua code can be reused to display the same infobox on any language version of Wikipedia.

The new system has now been deployed on several Gene Wiki pages within Wikipedia, and we will complete the migration after extensive testing and community consensus on all implementational details has been reached.

### Data usage beyond Wikipedia Gene Wiki infoboxes

**Figure 4:**
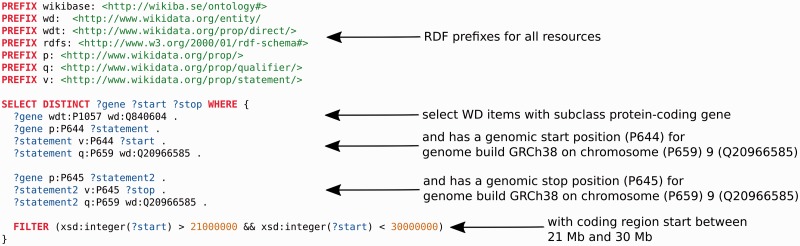
An example SPARQL query, using the Wikidata SPARQL endpoint (query.wikidata.org). It retrieves all Wikidata (WD) items which are of subclass protein-coding gene (Q840604), which have a chromosomal start position (P644) according to human genome build GRCh38 and reside on human chromosome (P659) 9 (Q20966585) and a chromosomal end position (P645) also on chromosome 9. Furthermore, the region of interest is restricted to a chromosomal start position between 21 and 30 megabase pairs. Colors: Red indicates SPARQL commands, blue represents variable names, green represents URIs and brown are strings. Arrows point to the source code the description applies to.

Data from Wikidata can be widely used in any application of interest. For example, the Gene Wiki infobox could now be rendered on any website on the Internet. As described in the introduction, a SPARQL endpoint, WDQ, the sophisticated Mediawiki API and a free text search engine constitute the main ways of querying data. These facilities can easily be integrated in downstream analysis using Python, R or any other data analysis language which supports seamless integration of data from the web. The SPARQL endpoint should be particularly useful for dynamic data integration. For example, for annotation of deletions in a cancer genome, it would be useful to know: ‘Which genes, encoding for proteins, are located on chromosome 9 between 21 and 30 megabase pairs?’. Content accessible through the Wikidata endpoint facilitates a single query that can answer this question immediately (Figure 4).

As the data are richly referenced, data origin and validity of statements can be reviewed instantly. Furthermore, the unique multilinguality of Wikidata, enabled by multilingual item labels, descriptions, and interwiki links to all of the Wikipedias allows the data to be used globally.

## Discussion

We created an open, community editable structured resource for all human and mouse genes and proteins using Wikidata as the technical platform. As a first use case of this data corpus, we demonstrated a remodelled Wikipedia Gene Wiki infobox which retrieves its data entirely from Wikidata, greatly simplifying the maintenance of these infoboxes. Until now, each of the 280 language-specific Wikipedias had to independently manage their infobox data in the context of MediaWiki templates meant for managing information display, not for storage and retrieval. This created significant redundancy, a great risk for errors and inconsistencies, and out of date data, as the same data needed to exist in every language-specific Wikipedia. Now, not only do the Wikipedias benefit from higher data quality when based on a centralized data repository, Wikidata also benefits from the focused human effort in the global Wikipedia community.

In addition to these benefits to the Wikipedia community, the Gene Wiki effort in Wikidata also offers many data integration advantages to the biomedical research community. Biomedical knowledge is fragmented across many resources and databases, and small-scale data integration is an often-repeated exercise in almost every data analysis project. As for the language-specific Wikipedias, these small-scale integration efforts are incomplete, inefficient and error-prone. Instead, users now have the option of accessing and querying a central biomedical resource within Wikidata that is already pre-populated with many key resources and identifiers. While we certainly recognize that our effort does not yet include every resource in the biomedical space, Wikidata does empower any user to contribute data from their resource of interest. For example, any user could easily contribute data from other third party resources [e.g. International Union of Basic and Clinical Pharmacology (IUPHAR) (14), DECIPHER (15), COSMIC (16)] with minimal effort. These contributions can range from programmatic addition of large databases, to the output of medium-sized biocuration efforts, to individual statements added by individual users. We consider integration of data on human and mouse genes and proteins as the basis also for our own efforts with Wikidata. Figure 4 illustrates how the data could be used by the scientific community. The query returns all protein coding genes on a certain region of human chromosome 9, the chromosomal coordinates being based on the human genome build GRCh38. As NCBI gene nor a different resource provide a SPARQL endpoint with access to genomic coordinates, to the best of our knowledge, a similar query cannot be achieved with any other semantic web resource currently available. As the Gene Ontology terms are attached to human proteins, a slightly modified query would also allow functional annotation of the gene products, e.g. for copy number aberrations of cancer genomes, through executing a SPARQL query. Currently, tools like ANNOVAR (17) provide richer annotation for variants and mutations, but Wikidata could e.g. prove useful for fast functional annotation of copy number aberrations in hereditary disease and cancer sequencing. With Wikidata growing, also the number of (annotation) use cases will increase. In addition to extending data in the biology and molecular biology space (e.g. cell lines, non-coding RNAs), we are currently importing data on human diseases and drugs.

An important aspect of the broad applicability and reusability of Wikidata is its connection to the Semantic Web and Linked Open Data (Figure 4). Wikidata IDs give genes and proteins stable Uniform Resource Identifiers (URIs) in the Semantic Web, which in turn link to other common identifiers used in the biomedical research community. Moreover, Wikidata provides perhaps the simplest interface for anyone to edit the Semantic Web, which is otherwise limited by high technical barriers to contribute. Similar or complementary efforts for integration of biomedical data are Bio2RDF and SNPedia. Bio2RDF integrates 37 different, well-established biomedical resources by defining an ontology for each data source and so converting them into normalized RDF documents (18, 19). The Bio2RDF data integration effort currently is considerably larger than our project. Apart from that, the data models in Wikidata are not based on ontologies, but they are a product of community discussion. Only the basic Wikidata data model exists as an ontology (https://www.mediawiki.org/wiki/Wikibase/Indexing/RDF_Dump_Format), which allows all data and data models in Wikidata to exist within this one ontology, which simplifies creation of new data models but might complicate semantic reasoning. Another major difference is that Wikidata can be edited by any user whereas for Bio2RDF, additions or corrections need to be performed by the maintainers. Furthermore, integration of smaller biomedical databases, which exist in large numbers (20), seems to be easier with Wikidata, whereas Bio2RDF currently might be better suited for dealing with very large numbers of triples. Although Bio2RDF users who would like to integrate their data sources of interest in their analysis still have the option to create their own Bio2RDF compatible RDF conversions, we think that having the data ready to go in a triple store should considerably simplify data analysis. Nevertheless, as both projects attempt to open biology to the semantic web, exploration of synergies between them seems worthwhile.

SNPedia is a semantic web enabled wiki for single nucleotide polymorphisms (SNPs) and also provides information on genes (21). Compared to Wikidata, SNPedia is restricted to SNPs and lacks a SPARQL endpoint or similar query capabilities. We do not have immediate plans to integrate SNP data, but we consider SNPedia a valuable resource which complements our efforts (22). Should SNP data be integrated into Wikidata, SNPedia would be either a data source or a resource Wikidata SNP items should be linking to.

Other wiki-based initiatives resembling our project, unfortunately seem to have ceased to exist (23), which might be due to difficulties establishing an active community. One major advantage of Wikidata is a large community with many contributors being experts in the field. Currently, Wikidata has >16 000 active users (https://www.wikidata.org/wiki/Special:Statistics).

Finally, Wikidata comes with a number of challenges relevant to the scientific community. The data model needs to be checked and protected against erroneous alterations, but true vandalism seems to be very rare. An one time import of data from a source is most likely not sustainable but the import of a certain data source should be a long term commitment to synchronization with the original source using bots. The community process might appear intimidating at first, but critical questions by the community help to improve one's approach. Moreover, who contributes, automatically becomes part of the community with the right to vote. Licensing of the original data source could also pose challenging for an import to Wikidata, because Wikidata content is licensed as public domain (CC0).

This work describes our initial effort to seed Wikidata with data from several key genomics resources. While this action has direct value to our Gene Wiki project, we hope and expect this first step to nucleate further growth of scientific data in Wikidata. With sufficient contribution and participation by the community, Wikidata can evolve into the most comprehensive, current and collaborative knowledge base for biomedical research.
